# Hand grenade blast injuries in the Eastern Democratic Republic of Congo: a case series of 38 patients

**DOI:** 10.1186/s12873-022-00599-4

**Published:** 2022-03-19

**Authors:** Paul Munguakonkwa Budema, Romeo Bujiriri Murhega, Tshibambe Nathanael Tshimbombu, Georges Kuyigwa Toha, Fabrice Gulimwentuga Cikomola, Paterne Safari Mudekereza, Léon-Emmanuel Mubenga, Ghislain Maheshe Balemba, Darck Cubaka Badesire, Ulrick Sidney Kanmounye

**Affiliations:** 1 Department of Surgery, Provincial General Reference Hospital of Bukavu, Bukavu, Democratic Republic of Congo; 2 Faculty of Medicine, Université Catholique de Bukavu, Bukavu, Democratic Republic of Congo; 3 Research Department, Association of Future African Neurosurgeons, Kinshasa, Democratic Republic of Congo; 4 Geisel School of Medicine at Dartmouth, 1 Rope Ferry Rd, Hanover, NH 03755 USA; 5 Department of Radiology, Provincial General Reference Hospital of Bukavu, Bukavu, Democratic Republic of Congo

**Keywords:** Conflict, Democratic Republic of Congo, Grenade injury, Trauma, Survival

## Abstract

**Introduction:**

The armed conflict in the Kivu province of the Democratic Republic of Congo has caused close to 12,000 deaths. One of the most lethal weapons in armed conflicts is the high explosive hand grenade. The study aimed to describe the epidemiology, presentation, and outcomes of hand grenade blast injuries (HGBI) in the Kivu province.

**Methods:**

In this case series, the authors present 2017 to 2020 HGBI admissions at a Congolese trauma center. Measures of central tendency and spread were computed for continuous data. Complication and mortality rates were equally computed. Admission-to-discharge data were disaggregated by the body part injured and by complication status and visualized using time-to-event curves.

**Results:**

Thirty-eight HGBI patients aged 31.4 (range 17–56) years were included in the study. Twenty-six (68.4%) were male and the patients were admitted 1.8 days post-injury on average. The patients were hemodynamically stable at admission; 84.2% received the antitetanic vaccine, 21.1% received broad-spectrum antibiotics, and all were debrided (100.0%). The complication rate was 13.2%, and the most common complication was anemia (7.9%). In addition, the mortality rate was 2.6%. The median admission-to-discharge time was 17.0 (range 4–71) days, and it was prolonged in patients with lower extremity injuries (23.0 days).

**Conclusion:**

HGBIs cause avertable death and disability in the Kivu regions. These data suggest that the burden of HGBIs can be reduced with appropriate preventive and health systems strengthening interventions.

## Introduction

### Definition

An explosive hand grenade is a detonating device that can be thrown by hand [1]. Explosive hand grenades should be distinguished from a rocket-propelled grenades which are projected at high speeds by a mechanical launcher [2]. Hand grenades can be defensive, offensive, or anti-tank. Defensive grenades are characterized by fragmentation, while offensive grenades have a high explosive charge [3].

Fragmentation shatters the grenade casing producing splinters (shrapnel) projected into the surrounding at high velocity and traveling up to 200 m from the detonation point [1]. An explosive is considered a high explosive based on the speed of the chemical reaction during the detonation, which is faster than the speed of sound in the material [4]. High explosive materials include composition C-4, trinitrotoluene (or TNT), dynamite, or acetone peroxide [4]. High explosive grenades are designed to stun their targets, usually in confined spaces [1, 3]. Of note, their fragments travel similar distances as those of fragmentation grenades [1]. Finally, anti-tank grenades are high explosive grenades with charges designed for heavy armored vehicles (warheads) [1, 3].

### Pathophysiology and management

Explosions create multiple blast waves. The most damaging of these waves is the first blast wave or shock wave [4]. The majority of the damage caused by the shockwave is due to the shock front. A shock front is composed of air traveling centrifugally and at supersonic speed from the blast epicenter [5]. Right after the shock front, a low-pressure superheated wind called the blast wind pulls the grenade debris back towards the center of detonation [5]. Additionally, the shock wave propels fragments from surrounding material (ex: shattered windows) at supersonic speeds inflicting further damage [4]. The shock wave may cause secondary fires that cause further injury to the victims [4]. Table 1 summarizes the mechanism of injury of explosive devices.

**Table 1:** Types of injury caused by explosive devices

Category	Characteristics
Primary	Caused by the shock wave
Secondary	Caused by high-velocity fragments (debris)
Tertiary	Caused by the projection of victims by the blast wind
Quarternary	All explosive-related injuries that do not fit into one of the previous categories

The air pressure fronts and fragments generated by explosions cause blunt and penetrating trauma. The most common blunt force injury from explosions is a blast injury, and the most lethal blast injury is the blast lung injury [6]. The burden of explosions is enormous because they affect multiple systems and multiple victims [6]. Their burden is further compounded by their rarity, making most civilian hospitals unfamiliar with their presentation. So they present challenges in triage, diagnosis, and management [6].

Hand grenades have a low mass and sectional density, leading to rapid loss of energy, deceleration of fragments, and poor penetration [7]. As a result, the distance between the injured body part and the detonation center is a predictor of injury severity. Body parts in contact with the hand grenade will sustain higher energy injuries resulting in wider and deeper lesions such as amputations, blast injuries to deep organs, and comminuted fractures [7].

Traumatic injuries of the extremities are less likely to cause death than traumatic injuries to the brain, thorax, and abdomen [8]. Shock waves preferentially injure air-filled organs like the lungs, middle ear, and abdomen [6]. The most feared complication of blast injuries, blast lung, can cause air emboli, acute respiratory distress syndrome, massive hemothoraces, and tension pneumothoraces [9]. Blast injuries present classically as a triad of apnea, bradycardia, and hypotension; however, they can be initially asymptomatic, only revealing themselves as late as 48 h post-injury [6]. A chest x-ray is recommended for all patients exposed to a blast injury [6, 10]. When blast lung injury is present, it should be managed with supplemental high flow oxygen, limiting the inspiratory pressure to < 40 cm H_2_O, drainage of blood and air collections in the pleural space, hyperbaric oxygen therapy (if air embolism), and permissive hypercapnia (if acute respiratory distress syndrome) [11].

### Context and study rationale

The Kivu conflict opposes the military forces of the Democratic Republic of Congo and the Democratic Forces for the Liberation of Rwanda [12]. Set in the North and South Kivu provinces of the Democratic Republic of Congo, the Kivu conflict has roots in the First Congo War of 1994 [12]. The conflict accounts for more than 13% of all traumatic injuries in the region [13] causing more than 12,000 deaths and leading to the internal migration of 1.4 million people [12]. Hand grenade blast injuries (HGBIs) are common in conflict-ridden regions but there are no studies reporting their prevalence in the Kivu regions [6].

The Congolese health system is organized into three administrative levels namely central, provincial, and peripheral. The central level is headed by the National Ministry of Health, the provincial level is headed by Provincial Ministries of Health, and the peripheral level is represented by the health facilities. From an administrative perspective, the Kivu conflict is overseen by two provincial Ministries of Health: North and South Kivu. The peripheral level is further organized into three levels: first, second, and third. The first level is represented by health centers and general regional hospitals. Provincial hospitals make up the second level while university hospitals make up the third level. Health centers offer primary care and are not equipped to manage traumatic injuries whereas general regional hospitals are designed to manage mild-to-moderate injuries. General regional hospitals have surgery and imaging units that are staffed mostly by general practitioners. The Congolese health system equally integrates private health facilities; the majority of which are faith-based [14] and other non-governmental organizations such as Medecins Sans Frontieres [13]. The hierarchical health system structure and lack of a formal emergency medical system are responsible for considerable informational lags between the peripheral and central levels that translate into delays in meeting urgent health needs.

This case report aimed to describe the landscape and outcomes of HGBIs in the largest trauma center of the South Kivu province between 2017 and 2020.

## Methods

### Study design and ethics

This study collected data on HGBI patients admitted to the emergency medicine, trauma, and neurosurgery departments of the Provincial General Reference Hospital of Bukavu, Bukavu, Democratic Republic of Congo. The study was authorized by the institutional review board of the same institution.

### Participants, study size, and data sources

Sociodemographic, clinical, therapeutic, and outcome data of HGBI patients admitted between January 1, 2017, and December 31, 2020, were extracted from paper patient medical records and stored in a Microsoft Excel spreadsheet (Microsoft, WA, USA). The cause of injury, HGBI, was ascertained using triangulation methods i.e., extracted from admission logbooks, medical records, and operative room logbooks. Patient data were collected by trained personnel using a standardized collection tool. Sociodemographic data included patient age, sex, and address. The distance from the hospital to the patient’s address was calculated using Google Maps (Google Inc., CA, USA) [15]. The clinical data included vital signs, SaO_2_, and injury patterns. The shock index was calculated by dividing the heart rate by the systolic blood pressure [16].

### Data analysis

SPSS Statistics v. 26 (IBM, New York, U.S.A.) was used for statistical analysis. Age, distance from the hospital, injury-to-admission time, vital signs, SaO_2_, hemoglobin concentration, and shock index were expressed as continuous variables. Continuous variables were summarized as means and measures of spread. In addition, the complication and mortality rates were calculated. Admission-to-discharge curves were plotted using a time-to-event function and the curves were disaggregated by the most injured body part and complication status.

## Results

One thousand and twenty-nine patients were injured by a firearm or hand grenade during the study period. Of these, 38 (3.7%) were injured by a hand grenade (Table 2). There were two patients (5.3%) in 2017, 26 (68.4%) in 2018, eight (21.1%) in 2019, and two (5.3%) in 2020. The majority were male (*n* = 26, 68.4%), and eight (21.1%) had an open fracture. Of the eight patients with open fractures, five had lower limb fractures, while three had upper limb fractures.

**Table 2:** Descriptive presentation of hand grenade blast injury patients in the Eastern Democratic Republic of Congo

ID	Age (Years)	Sex	Injured body part
1	24	M	Penetrating injury to the left thigh and right leg
2	50	F	Transfixing injury to the left shoulder and right forearm
3	22	M	Multiple gluteal, lumbar, and thoracic penetrating injuries
4	43	M	Penetrating injury to the mandible and right leg
5	49	F	Multiple penetrating injuries to the right hemibody
6	30	M	Penetrating injury to the left elbow and right leg
7	45	M	Penetrating injury to the right thigh and left leg
8	36	M	Multiple penetrating injuries to the left hemibody
9	20	M	Incomplete amputation of the right hand
10	32	M	Penetrating injury to the left thorax, abdomen, and hand
11	7	F	Penetrating injury to the right hand
12	48	M	Penetrating injury to the left eye
13	30	M	Amputation of 4 left hand fingers and penetrating injury to the thorax and abdomen
14	28	M	Penetrating injury to the left hand and face
15	33	M	Penetrating injury to the face and thorax
16	19	F	Penetrating injury to the right thigh and leg
17	17	M	Penetrating injury to the left ankle
18	39	F	Penetrating injury to the left scapula and forearm
19	20	M	Penetrating injury to the right temporoparietal skull, arm, forearm, and buttock
20	50	F	Penetrating injury to the left abdomen, shoulder, and forearm
21	40	F	Penetrating injury to the left back, buttock, and foot
22	22	M	Penetrating injury to the left calf, and right arm, thigh, and leg
23	28	F	Penetrating injury to the left hand and thigh
24	22	F	Penetrating injury to both legs
25	23	M	Penetrating injury to the abdomen and right hand
26	22	M	Penetrating injury to the left buttock
27	50	F	Penetrating injury to the right thorax, abdomen, and ankle
28	42	M	Penetrating injury to the right thigh
29	24	M	Penetrating injury to the left thorax, hand, and thigh
30	38	F	Penetrating injury to the left neck
31	15	M	Penetrating craniocerebral injury and penetrating injury to the forearm
32	32	M	Penetrating injury to the right thigh
33	23	M	Penetrating injury to the left leg
34	52	M	Penetrating injury to the face, right thigh, and left leg
35	8	M	Penetrating injury to the left arm
36	6	M	Penetrating injury to the right thorax
37	56	F	Penetrating injury to the left knee
38	38	M	Penetrating craniocerebral injury

HGBI patients were admitted at our facility 1.8 (SD 3.8) days post-injury. At admission, most had normal heart rate (SD 13.0) bpm, respiratory rate (SD 2.9) cpm, and hemoglobin concentration (SD 2.0) g/dL (Table 3).

**Table 3:** Mean, 95% confidence interval, and standard deviation values of quantitative clinical parameters

Characteristic	Mean	95% CI	Standard Deviation
Age (years)	31.4	26.9–35.9	11.9
Distance from the hospital (km)	70.7	26.9–114.5	135.9
Injury-to-admission time (days)	1.8	0.6–3.1	3.8
Heart rate (bpm)	90.7	85.7–95.6	13.0
Systolic blood pressure (mmHg)	98.6	78.1–119.0	15.2
Diastolic blood pressure (mmHg)	60.0	43.4–76.5	14.4
Respiratory rate (cpm)	22.2	21.2–23.2	2.9
Temperature (°C)	36.5	36.3–36.7	0.6
SaO_2_ (%)	65.8	41.3–90.3	2.2
Hemoglobin concentration (g/dL)	12.1	11.3–12.8	2.0
Shock index	0.82	0.74–0.90	0.21

Thirty-two (84.2%) patients were vaccinated against tetanus, eight (21.1%) were given broad-spectrum antibiotics, six (15.8%) were given gentamicin, and three (7.9%) were given metronidazole.

All patients had a debridement (*n* = 38, 100.0%), two (5.3%) had an amputation, and two (5.3%) had a disarticulation. Nine patients (23.7%) needed multiple debridements: six (15.8%) needed two sessions, two (5.3%) needed three, and one (2.6%) needed four sessions.

Three patients (7.9%) developed anemia, while one patient (2.6%) developed a pulmonary contusion, and another developed a tension pneumothorax. None of the patients developed an infection, and none of the patients with complications had an open fracture. The patient with a tension pneumothorax died (2.6%).

**Fig. 1:**
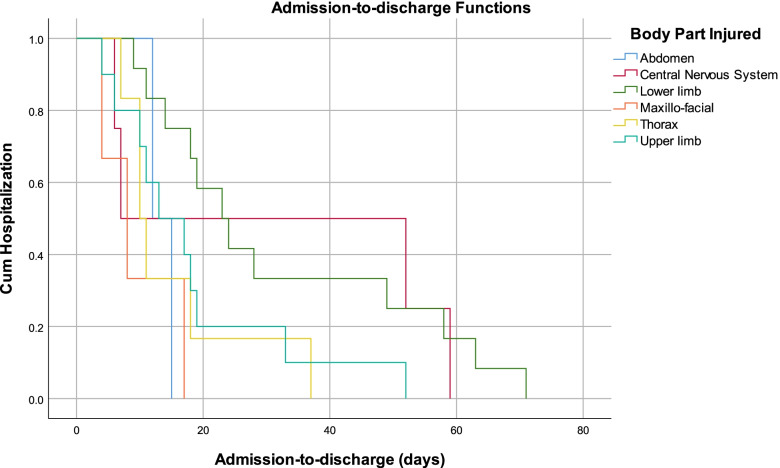
Admission-to-discharge curves disaggregated by the most injured body part injured

**Fig. 2:**
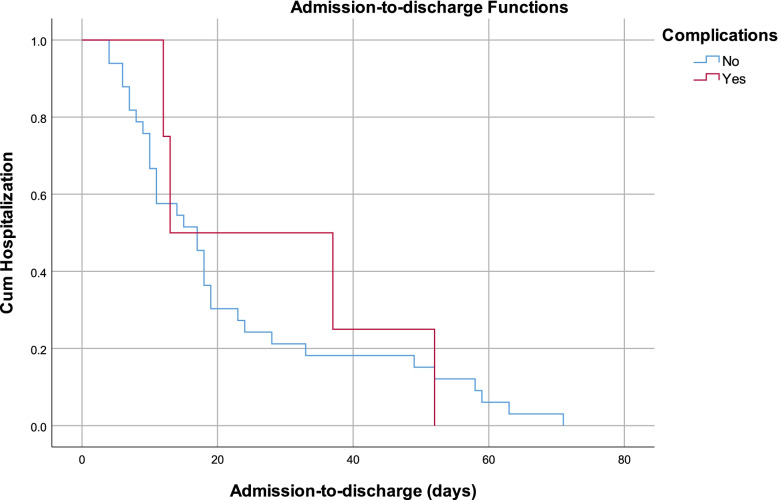
Admission-to-discharge curves disaggregated by the complication status

The overall median admission-to-discharge time was 17.0 (*n* = 38, mean 22.8, SE = 3.1) days. Patients with lower limb injuries had the longest median admission-to-discharge time, 23.0 (*n* = 12, mean 32.3, SE = 6.3) days, while patients with central nervous system injuries had the shortest median admission-to-discharge time 7.0 (*n* = 4, mean 31.0, SE = 14.2) days (Fig. 1). Patients who did not experience complications had longer median admission-to-discharge times (*n* = 33, 17.0 days, mean 22.1 days, SE = 3.3 vs. *n* = 5, 13.0 days, mean 28.5 days, SE = 9.7) (Fig. 2).

## Discussion

This is the first study to describe HGBI in the Democratic Republic of Congo. HGBI affects young adult males disproportionately, and patients travel long distances to get care at the authors’ institution. Most HGBI patients have borderline blood pressure values and a shock index suggestive of hypovolemic shock. The most common complication is anemia, and the majority of patients have nonfatal HGBI.

The majority of HGBI patients were young adult males. This subpopulation is more likely to be affected by HGBIs because young adult males are often involved in armed conflicts [12]. When they are not participating in the conflict, young adults can still be exposed to HGBIs if they have an outdoor occupation [17]. This is especially true if they are from a low socioeconomic background. The authors’ institution is located in the capital of South Kivu, Bukavu, and the majority of patients lived between 27 and 115 km away from the hospital. The closest urban center to Bukavu is Goma, a city 195 km away [18]. As a result, the majority of HGBI lived in rural areas around Bukavu and were probably from a low socio-economic background. Five of the Congolese HGBI patients were minors. Children are more likely to exhibit unsafe behavior around hand grenades, including approaching, touching, and playing with hand grenades [17, 19]. This at-risk behavior equally increases their chances of severe injury. Between 1998 and 2007, 13 of the 21 civilian HGBI fatalities in Transkei (South Africa) were children [19]. Hence, children in high-risk areas should be trained to recognize, report, and avoid playing with or around hand grenades.

HGBI patients get to the definitive trauma care facility more than an hour after the injury, and severely injured patients arrive later than less severely injured patients (equally known as the *upside-down triage*) [6]. This finding highlights barriers in getting timely definitive care and can be traced back to the Congolese health system organization. Half of HGBI patients were discharged more than 17 days after their admission, and the median length of stay was longer for patients with lower limb injuries. The majority of open fractures affected the lower limbs. These injuries require surgical management and longer hospital stays [20].

This study has multiple limitations including monocentric data, a small sample size, and lack of follow-up. The small sample size is a consequence of the rarity of HGBIs, and the lack of follow-up data is due to patients living far away from the trauma center. Patients who come to the hospital for follow-up visits bear the cost of transport out-of-pocket and face financial risk from lost wages. These are significant causes of loss to follow-up in low-resource settings [21]. Next, this study reports descriptive in-hospital data which cannot be extrapolated to the population-level without making multiple assumptions. Data collection was complicated by two factors. First, the Democratic Republic of Congo lacks a structured referral system and next the Kivu Conflict is geographically and temporally complex. The Kivu Conflict is set in two regions: North Kivu (Area: 59,483 km^2^, Population: 6.655 million) and South Kivu (Area: 65,070 km^2^, Population: 5.772 million). Moreover, the conflict is fought between numerous parties including the DRC national army, Rwandan forces, UN forces, and militias (M23, FDLR, RUD-Urunana, FNL-Nzabampema, FPB, NDC, etc.) on multiple battlegrounds. As a result, the obtention of more granular pre-hospital data is difficult. Of note, this study reports the experience of the largest trauma center in the two Kivu regions. This trauma center is located in a university hospital and is supported by the International Committee of the Red Cross. The International Committee of the Red Cross reduces transportation times by airlifting patients from its outposts to the trauma center. In addition, the Committee covers medical expenses incurred during the patients’ hospitalization. The Medecins Sans Frontieres outpost in the North Kivu has a significantly smaller surgical volume than the authors’ institution and has reported an almost inexistent HGBI prevalence [13]. This finding supports the belief that the authors’ institution manages a substantial proportion of trauma patients in this region.

Notwithstanding the aforementioned limitations, this study presents a comprehensive overview of HGBIs in the Eastern Democratic of Congo from 2017 to 2020.

## Conclusion

This in-hospital study suggests that HGBIs cause a significant burden in the Kivu provinces. Additionally, the annual incidence of HGBI has been constant except for an increase in 2018. Young adult Congolese males are the most affected, and the majority of patients are admitted to the authors’ facility more than 1 day after their injury. The complication rate was high; however, the authors did not register infections. Predictably, one patient died from a tension pneumothorax. Furthermore, patients with lower extremity injuries and those who did not experience complications had longer lengths of stay than their counterparts. Moving forward, research in this area should focus on identifying the correlates of complication and mortality. In addition, public health interventions are needed to reduce the injury-to-admission time among HGBI patients. Belligerent and neutral parties must convene to discuss a ceasefire. Finally, it is essential that stakeholders invest in more robust information management structures to collect disaggregated epidemiological data from the battlefield.

## Data Availability

The datasets used and/or analysed during the current study are available from the corresponding author on reasonable request.
